# Functional role of ambient GABA in refining neuronal circuits early in postnatal development

**DOI:** 10.3389/fncir.2013.00136

**Published:** 2013-08-13

**Authors:** Giada Cellot, Enrico Cherubini

**Affiliations:** Department of Neuroscience Scuola Internazionale Superiore di Studi AvanzatiTrieste, Italy

**Keywords:** development, hippocampus, tonic GABAA conductance, network activity, extrasynaptic GABA_A_ receptor

## Abstract

Early in development, γ-aminobutyric acid (GABA), the primary inhibitory neurotransmitter in the mature brain, depolarizes and excites targeted neurons by an outwardly directed flux of chloride, resulting from the peculiar balance between the cation-chloride importer NKCC1 and the extruder KCC2. The low expression of KCC2 at birth leads to accumulation of chloride inside the cell and to the equilibrium potential for chloride positive respect to the resting membrane potential. GABA exerts its action *via* synaptic and extrasynaptic GABA_A_ receptors mediating phasic and tonic inhibition, respectively. Here, recent data on the contribution of “ambient” GABA to the refinement of neuronal circuits in the immature brain have been reviewed. In particular, we focus on the hippocampus, where, prior to the formation of conventional synapses, GABA released from growth cones and astrocytes in a calcium- and SNARE (soluble *N*-ethylmaleimide-sensitive-factor attachment protein receptor)-independent way, diffuses away to activate in a paracrine fashion extrasynaptic receptors localized on distal neurons. The transient increase in intracellular calcium following the depolarizing action of GABA leads to inhibition of DNA synthesis and cell proliferation. Tonic GABA exerts also a chemotropic action on cell migration. Later on, when synapses are formed, GABA spilled out from neighboring synapses, acting mainly on extrasynaptic α5, β2, β3, and γ containing GABA_A_ receptor subunits, provides the membrane depolarization necessary for principal cells to reach the window where intrinsic bursts are generated. These are instrumental in triggering calcium transients associated with network-driven giant depolarizing potentials which act as coincident detector signals to enhance synaptic efficacy at emerging GABAergic and glutamatergic synapses.

γ-aminobutyric acidergic (GABAergic) signaling plays a crucial role for processing and storage of information in the brain. By releasing γ-aminobutyric acid (GABA) into distinct targeted subcellular compartments, GABAergic interneurons regulate cells excitability and dictate the temporal dynamics of principal cells firing giving rise to networks oscillations thought to support distinct brain states and high cognitive functions (Klausberger and Somogyi, 2008). The action of GABA relies on the temporally and spatially regulated expression of GABA_A_ receptors which mediate two distinct forms of inhibition: phasic and tonic (Semyanov et al., 2004; Cavelier et al., 2005; Farrant and Nusser, 2005). Phasic inhibition is mediated by local release of GABA from presynaptic vesicles (Cherubini, 2012). Once released, GABA binds to synaptic GABA_A_ receptors facing presynaptic release sites and trigger fast inhibitory postsynaptic potentials (IPSPs), regulating point-to-point communication between neurons. In this case, synaptic GABA_A_ receptors are exposed for a very short period of time to high concentrations of GABA. GABA diffuses throughout the neuropil before being taken up by selective plasma membrane transporters, which contribute to the clearance of the neurotransmitter and to shape synaptic currents (Cherubini and Conti, 2001). This transient inhibitory action is important for timing-based signaling, setting the temporal window for synaptic integration (Pouille and Scanziani, 2001) and synchronization of neuronal networks (Cobb et al., 1995).

Tonic inhibition is mediated by “ambient” GABA originated from spillover of the neurotransmitter escaping the synaptic cleft (Kaneda et al., 1995; Brickley et al., 1996; Wall and Usowicz, 1997), from astrocytes *via* a non-vesicular calcium-independent process (Liu et al., 2000; Schousboe, 2003) or from the reversed transport (Attwell et al., 1993; Wu et al., 2001). In all these cases extrasynaptic GABA_A_ receptors are persistently exposed to submicromolar concentrations of GABA present in the extracellular space. This requires extrasynaptic GABA_A_ receptors with high affinity for GABA and relatively insensitive to desensitization. Selective plasma membrane transporters contribute to the clearance of GABA thus regulating its concentration in the extracellular space, in particular during massive release (Bragina et al., 2008). The resulting GABA-mediated tonic conductance is involved in regulating network excitability, cell firing and oscillatory behavior. In addition, the persistent increase in tonic conductance may affect the magnitude and duration of voltage responses to injected currents and increase the decrement of voltage with distance (Farrant and Nusser, 2005).

Synaptic and extrasynaptic GABA_A_ receptors are thought to belong to separate entities since they appear to be composed of different subunits. However, the introduction of single molecule imaging technique has enabled measuring individual receptor movements in the plane of the plasma membrane (Triller and Choquet, 2005; Jacob et al., 2008; Luscher et al., 2011). This approach has revealed that receptors undergo lateral diffusion that allows them to continuously exchange between synaptic and extrasynaptic sites. Most receptors are delivered to extrasynaptic locations from where they can move and be trapped into synapses. GABA_A_ receptors trafficking and clustering is regulated by the scaffold protein gephyrin which, by anchoring GABA_A_ receptors to the cytoskeleton, exerts a stabilizing action.

The main focus of this review is on the contribution of “ambient” GABA in sculpting neuronal circuits at early developmental stages. We will first provide a brief overview of the depolarizing and excitatory action of GABA during embryonic and early postnatal life, emphasizing the role of this neurotransmitter in controlling cells proliferation, growth, migration, and differentiation during cortical neurogenesis as well as synaptogenesis immediately after birth. Then, we will discuss how a persistent tonic GABA_A_-mediated conductance is instrumental in increasing cell excitability, thus contributing to trigger network-driven giant depolarizing potentials or GDPs in the immature hippocampus. GDPs are known to act as coincidence detectors for enhancing synaptic efficacy at emerging glutamatergic and GABAergic synapses. Finally, we will discuss how, immediately after birth, ambient GABA regulates cell excitability in other brain structures.

## AT EARLY DEVELOPMENTAL STAGES GABA DEPOLARIZES AND EXCITES TARGETED CELLS *VIA* AN OUTFLUX OF CHLORIDE

**FIGURE 1:**
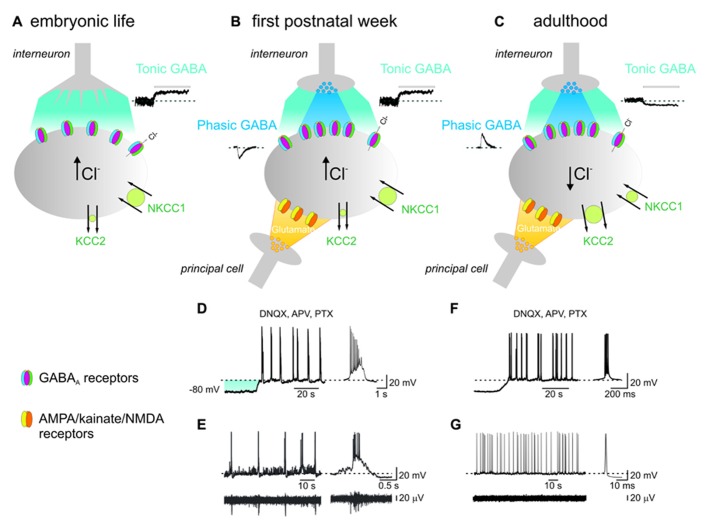
**In the immature hippocampus, ambient GABA depolarizes targeted cells and contributes to generate network-driven GDPs.** **(A)** Late in embryonic, early in postnatal life, prior to synapses formation, GABA released from growth cones diffuses in the extracellular space (light blue), binds to GABA_A_ receptors located on the membrane of a neighboring cell (gray) and depolarizes the membrane through an outwardly directed flux of chloride. This results from the peculiar balance between the cation-chloride importer NKCC1 (large circle in green) and the poorly expressed cation-chloride extruder KCC2 (small circle in green), leading to accumulation of chloride inside the cell [Cl^-^]_i_. Tonic GABA current can be unveiled by applying picrotoxin (PTX, 100 μM; bar) as illustrated in the inset on the right. **(B)** After birth, during the first postnatal week, chemical GABAergic and glutamatergic synapses start appearing. GABA released by exocytosis from presynaptic vesicles (dark blue) acts on postsynaptic GABA_A_ receptors located in face to presynaptic release sites and generate synaptic currents (inset on left). Once released GABA spills out (light blue) to activate extrasynaptic GABA_A_ receptors. At this stage, glutamatergic synapses are also formed (yellow). The synergistic action of GABA and glutamate, both depolarizing and excitatory is crucial for GDPs generation. At this developmental stage, both phasic and tonic GABA are depolarizing since the cation-chloride extruder KCC2 is still poorly expressed on the membrane surface (small circle in green). **(C)** In adulthood, GABA acting on both synaptic and extrasynaptic GABA_A_ receptors hyperpolarizes the membrane. This occurs because, due to the developmental up-regulation of the cation-chloride extruder KCC2 expression (large circle in green), [Cl^-^]_i_ is maintained at very low levels and when GABA opens GABA_A_ receptor channels, causes a net flux of chloride inside the cell leading to membrane hyperpolarization and inhibition of cell firing. In addition, the concomitant down-regulation of the cation-chloride importer NKCC1 with age (small circle in green), contributes to maintain a very low [Cl^-^]_i_. Note the opposite direction of both phasic (inset on left) and tonic (inset on the right) GABA and the reduced amplitude of the latter respect to neonates. **(D)** During the first postnatal week, depolarizing the membrane in the presence of AMPA/kainate/NMDA and GABA_A_ receptor antagonists (DNQX, APV, and PTX), induces the appearance of intrinsic voltage-dependent bursts which are instrumental in triggering GDPs (see text). On the right a single burst recorded on an expanded time scale. The shadow (light blue) represents the membrane depolarization induced by tonic GABA. **(E)** Individual whole cell (neonatal CA3 pyramidal neuron; upper trace) and concomitant extracellular field recordings of spontaneous network-driven GDPs (bottom trace). On the right a single GDP and a concomitant field potential are represented on an expanded time scale. **(F)** Intrinsic bursts induced in adulthood by depolarizing the membrane in the presence of DNQX, APV, and PTX. Note the difference in burst duration between adults and neonates. **(G)** Whole cell (upper trace) and concomitant extracellular recordings (bottom trace) obtained from an adult CA3 pyramidal cell. On the right a single spike is shown on an expanded time scale. Note the absence of network-driven correlated activity such as GDPs (**D** and **F** modified from Safiulina et al., 2008; **E** modified from Ben-Ari et al., 2007).

GABAergic signaling is unique in that the polarity of its action largely depends on the intracellular chloride concentration [Cl^-^]_i_, leading in certain conditions to depolarizing and even excitatory effects. Neuronal [Cl^-^]_i_ is under the control of cation-chloride co-transporters (CCCs), intrinsic membrane proteins that transport Cl^-^ ions, together with Na^+^ and/or K^+^ ions, in an electroneutral manner due to the stoichiometric coupling and directionality of translocated ions. The two main CCCs which control chloride concentration inside the cell are the Na–K–2Cl importer NKCC1 and the K–Cl extruder KCC2. The low expression of KCC2 at birth leads to accumulation of chloride inside the cell and to the equilibrium potential for chloride (*E*_Cl_^-^) positive respect to the resting membrane potential (*V*_m_). The progressive reduction in [Cl^-^]_i_ with age, due to the developmentally up-regulated expression of KCC2 and the concomitant down-regulated expression of NKCC1 (Yamada et al., 2004; Dzhala et al., 2005), leads to relatively low [Cl^-^]_i_ (*E*_Cl_^-^ close to *V*_m_; **Figure 1**; Rivera et al., 1999; Blaesse et al., 2009). Hence, while in adulthood GABA released from local interneurons opens GABA_A_ receptor channels causing a net flux of chloride inside the cells with consequent membrane hyperpolarization and reduction of cell firing (Cherubini and Conti, 2001), in the immediate postnatal period it depolarizes the membrane of targeted cells through an outwardly directed flux of chloride (Ben-Ari et al., 1989, 2012; Cherubini et al., 1991), thus enabling the membrane to reach spike threshold *via* amplification through a persistent non-inactivating sodium conductance (Valeeva et al., 2010; see also Song et al., 2011). It is worth noting that GABA can depolarize and still inhibit targeted cells *via* its shunting action (Mohajerani and Cherubini, 2005; Banke and McBain, 2006).

γ-aminobutyric acid-induced membrane depolarization activates voltage-dependent calcium channels and removes the voltage-dependent magnesium block from *N*-methyl-D-aspartic acid (NMDA) receptors leading to large calcium influx (Ben-Ari et al., 1997; Leinekugel et al., 1997). Calcium entry is crucial for the activation of second messengers, involved in a variety of developmental processes, from cell migration and differentiation to synaptogenesis.

Interestingly, GABAergic signals can be shifted in polarity by activity. This unique form of plasticity involves changes in the expression of CCCs able to convert GABA responses from hyperpolarizing to depolarizing and *vice versa *(Fiumelli and Woodin, 2007). While in adult hippocampal neurons, coincident detection of pre and postsynaptic signals alters the activity of KCC2 leading to changes in *E*_Cl_ and in the strength of inhibition (Woodin et al., 2003), in immature cells modifies the expression of NKCC1 causing *E*_Cl_ to shift toward more negative values (Balena and Woodin, 2008). Therefore, the dynamic regulation of intracellular chloride strictly depends on the ongoing activity generated by neurons and this may affect not only synaptic but also extrasynaptic GABA_A_ receptors.

Synapses start developing from birth following a well-defined sequence of events: GABAergic signals develop before glutamatergic ones whose operation correlates with the level of dendritic arborization (Tyzio et al., 1999; Ben-Ari et al., 2007). This occurs first in interneurons, and then in principal cells, indicating that GABAergic interneurons provide the early source of activity in otherwise silent networks (Gozlan and Ben-Ari, 2003).

Interestingly, prior to the formation of conventional synapses, GABA released from growth cones and astrocytes in a calcium- and soluble N-ethylmaleimide-sensitive-factor attachment protein receptor (SNARE)-independent way, diffuses away to activate in a paracrine fashion extrasynaptic receptors (**Figure 1**; Demarque et al., 2002). These are expressed at very early stages of development by neuronal precursors and by neurons in several brain areas giving rise to a tonic conductance (Nguyen et al., 2001). The existence of such conductance can be estimated by the shift in the baseline current obtained by blocking GABA_A_ receptors with selective antagonists. The tonic current persists after treatment with calcium channel blockers or botulin toxin which cleaves SNAP-25 (synaptosomal-associated protein 25), a SNARE protein that prevents vesicular release. In addition, a tonic current is present in Munc18-1 deficient mice lacking vesicular release (Demarque et al., 2002). It has been proposed that a relatively poor clearance system (but see Sipilä et al., 2004; Safiulina et al., 2006) enables GABA to accumulate in the extracellular space and reach a concentration sufficient to exert its depolarizing and excitatory effects on distal neurons making this form of intercellular communication very effective. In addition, since the main neuronal GABA transporter GAT-1 carries chloride along with GABA and sodium, the possibility that a high intracellular chloride may decrease the efficacy of GABA uptake cannot be excluded.

## AMBIENT GABA REGULATES CELL MIGRATION AND SYNAPSES FORMATION

The construction of the cerebral cortex from a single sheet of neuroepithelium relies on a sequence of well-orchestrated developmental processes. Neurons must be generated in the correct number, migrate to the proper position, and form connections with neighboring cells. Of the many cell-intrinsic and -extrinsic signals involved in neocortical development, ambient GABA plays a central role in these processes (Wang and Kriegstein, 2009).

During corticogenesis, endogenous GABA present in the extracellular space, binds with high affinity (higher than in postmigratory neurons) to extrasynaptic GABA_A_ receptors (relatively insensitive to desensitization), expressed on migrating neurons as well as on radial glia and causes a membrane depolarization and a transient increase in calcium *via* voltage-dependent calcium channels. This leads to inhibition of DNA synthesis and cell proliferation as assessed by the reduced number of progenitors incorporating BrdU (LoTurco et al., 1995; Owens and Kriegstein, 2002). Furthermore, tonic GABA exerts a chemotropic action on cell migration as demonstrated by the observation that blocking GABA_A_ receptors with bicuculline in organotypic hippocampal slices reduces the migration of neuroblasts (Manent et al., 2005). Ambient GABA may also regulate the speed of migration of young neurons in the rostral migratory stream (Bolteus and Bordey, 2004) and provide a stop signal for ending migration (Behar et al., 2000). The effect of GABA on cell migration has been recently questioned. Using *in utero* electroporation, Cancedda et al. (2007) have clearly demonstrated that the premature expression of the cation-chloride extruder KCC2, which eliminates the depolarizing action of endogenous GABA in a subpopulation of newly born cortical neurons, does not alter their migration while at P4–P6 severely affects their morphological structure (neurons exhibit few short dendrites). However, as highlighted in a recent study (Inoue et al., 2012), the ectopic expression of KCC2 is functional only in the postnatal but not in the embryonic brain. This is probably related to endogenous taurine, particularly abundant in the fetal brain that, *via* the with-no-lysine protein kinase 1 (WNK1) signaling pathway, exerts an inhibitory action on KCC2 activation.

Increasing evidence suggests that a tonic GABA_A_-mediated membrane depolarization provides the first excitatory drive necessary for promoting neurite outgrowth and synapse formation. Unlike glutamate in fact, GABA depolarizes and at the same time, by clamping the membrane potential close to *E*_Cl_ (in immature neurons about -40 mV) exerts a shunting effect that would prevent an excessive calcium entry through voltage-dependent calcium channels with consequent excitotoxicity (Safiulina et al., 2010). GABA-mediated membrane depolarization has been shown to regulate the formation of glutamatergic synapses in the developing cortex *in vivo*, an effect that needs the contribution of NMDA receptors. This will allow a proper balance between excitation and inhibition, essential for the correct functioning of neuronal circuits (Wang and Kriegstein, 2008). Cortical neurons begin to express NMDA receptors during migration to the cortical plate. However, these receptors do not conduct at rest because they are blocked by magnesium. By facilitating the relief of the voltage-dependent magnesium block, GABA *via* its depolarizing action renders these receptors conductive. The systemic blockade of early GABA-mediated depolarization during a critical period between E17-P7 in mice with bumetanide, a selective NKCC1 inhibitor, leads to lasting disruption of AMPA receptors-mediated glutamatergic transmission in the adult cortex and to an excitatory/inhibitory imbalance (Wang and Kriegstein, 2011). Morphological analysis of bumetanide-treated mice revealed reduced spines density and dendritic arborization in cortical neurons (Wang and Kriegstein, 2011). This is in contrast with the data obtained by Pfeffer et al. (2009) from *Nkcc1*^-/-^ mice. Compensatory mechanisms responsible for the slightly depolarizing action of GABA in the genetic model may account for this discrepancy (Sipilä et al., 2009).

Interestingly, in the adult brain, GABA-mediated tonic excitation drives synaptic integration of newly generated neurons in pre-existing functional circuits suggesting that adult neurogenesis recapitulates the sequence of events occurring in immature cells at embryonic and early stages of postnatal development (Ge et al., 2006). As in postnatal development, conversion of GABA-induced depolarization into hyperpolarization in newborn dentate gyrus cells leads to significant defects in the formation of GABAergic and glutamatergic synapse as well as in dendritic arborization (Ge et al., 2006).

## IMMEDIATELY AFTER BIRTH, A TONIC GABA_A_-MEDIATED CONDUCTANCE CONTRIBUTES TO THE TRIGGERING OF NETWORK-DRIVEN GDPs IN THE HIPPOCAMPUS

In the neonatal hippocampus a GABA_A_ receptor-mediated tonic conductance has been well-characterized in both CA1 (Demarque et al., 2002; Marchionni et al., 2007) and CA3 regions (Marchionni et al., 2007; Sipilä et al., 2007). In these areas, principal cells exhibit a sustained tonic conductance (larger in the CA3 region; Marchionni et al., 2007) which plays a crucial role in enhancing cell excitability and neuronal firing, thus contributing to GDPs generation (Sipilä et al., 2005, 2009).

Tonic GABA_A_-mediated currents are more pronounced in neonates than in adults (**Figure 1**; Stell and Mody, 2002; Semyanov et al., 2003; Stell et al., 2003; Caraiscos et al., 2004). This has been attributed to the relatively poor GABA clearance caused by the low expression of GABA transporters. However, at least GAT-1 has been found to be present and functional from birth as shown by the ability of NO-711, a selective GAT-1 inhibitor, to enhance the decay kinetics of GABA_A_-mediated synaptic currents (Safiulina et al., 2006) and the duration of GDPs (Sipilä et al., 2004), indicating that GABA uptake controls GABAergic transmission and limits the action of GABA on GDPs. It is worth mentioning that while NO-711 affects the shape of synaptic currents (Safiulina et al., 2006), it only slightly alters tonic currents (Marchionni et al., 2007; Sipilä et al., 2007), suggesting that GAT-1, due to its preferential localization on axon terminals (Minelli et al., 1995), is more efficient at removing GABA from synapses than from the extracellular space.

In both CA1 and CA3 principal cells, “ambient” GABA may originate at least in part from spillover of the neurotransmitter from neighboring synapses during activity as shown by the reduction in the tonic current by tetrodotoxin (TTX) that blocks sodium currents and propagated action potentials (Marchionni et al., 2007; but see Sipilä et al., 2007 for the CA3 region). Interestingly, in contrast with adult guinea pigs (Semyanov et al., 2003) or rats (Marchionni et al., 2007), in neonatal animals stratum radiatum GABAergic interneurons fail to exhibit any sustained background conductance. This may be due either to a more efficient uptake system, able to maintain extracellular GABA at very low levels, or to a low expression of extrasynaptic receptors able to sense GABA. While the first possibility is unlikely since NO-711 did not modify the holding current, the second one remains to be demonstrated. Whether other transporters, different from GAT-1 are actively involved in removing GABA from GABAergic interneurons cannot be excluded.

In immature CA1 and CA3 principal cells, the selective use of GABA_A_ receptor agonists and antagonist has unveiled the presence of various extrasynaptic receptor subunits. These include the α1, α3 (Sipilä et al., 2007), α5, β2, β3, and γ2 (Marchionni et al., 2007). While α5 GABA_A_ receptor subunits are highly expressed in the neonatal hippocampus (Laurie et al., 1992; Didelon et al., 2000), δ subunits are relatively scarce (Didelon et al., 2000) and this may explain why allotetrahydrodeoxycorticosterone (THDOC), α δ subunit selective neuroactive steroid (Korpi and Lüddens, 1997), was unable to modify the baseline current (Marchionni et al., 2007).

The γ2, α1, and α3 subunits are crucial for zolpidem sensitivity. The observation that zolpidem potentiates tonic currents only in neonates (Sipilä et al., 2007) but not in adults (Semyanov et al., 2003) suggests a different expression of extrasynaptic GABA_A_ receptor subunits with age.

How could the tonic GABA_A_-mediated conductance influence network activity immediately after birth? Thanks to the depolarizing and excitatory action of GABA at this stage of development the tonic conductance has been found to enhance pyramidal cells excitability and to reduce the threshold for action potential generation (Marchionni et al., 2007). Thus, in cell-attach experiments to maintain intact the intracellular chloride concentration, picrotoxin blocks synaptic and extrasynaptic GABA_A_ receptors leading to an increased firing in pyramidal cells but not in interneurons. This may enhance glutamate release from principal cells.

Giant depolarizing potentials, which represent a primordial form of synchrony between neurons, are generated by the synergistic action of glutamate and GABA, both of which are depolarizing and excitatory (**Figure 1**; Cherubini et al., 1991; Ben-Ari, 2002). In analogy with the synchronized activity generated in the disinhibited hippocampus (De la Prida et al., 2006), GDPs emerge when a sufficient number of cells fire and the excitability of the network attains a certain threshold within a restricted period of time. Although the entire hippocampal network possesses the capacity to generate GDPs, the CA3 area is centrally involved because: (i) in this area GABAergic interneurons with large axonal arborizations operate as functional hubs to synchronize large ensembles of cells (Bonifazi et al., 2009; Picardo et al., 2011; Allene et al., 2012); (ii) principal cells are connected by extensive glutamatergic recurrent collaterals; (iii) principal cells give rise to intrinsic bursts that drive other neurons to fire (Sipilä et al., 2005; Safiulina et al., 2008). In this context, GABA_A_-mediated tonic inhibition provides the membrane depolarization needed for principal cells to reach the window where intrinsic bursts are generated (**Figure 1**; Sipilä et al., 2007; Ben-Ari et al., 2012).

Since GDPs are instrumental for enhancing synaptic efficacy at emerging glutamatergic and GABAergic synapses and for converting silent synapses into conductive ones (Kasyanov et al., 2004; Mohajerani et al., 2007), we can speculate that, immediately after birth, ambient GABA exerts a crucial role in synaptic wiring. Whether changes in synaptic efficacy are associated with structural modifications necessary to rewire local neuronal circuits remain to be demonstrated.

## EARLY IN POSTNATAL DEVELOPMENT, TONIC GABAergic CURRENTS DIFFERENTLY REGULATES CELL EXCITABILITY IN VARIOUS BRAIN REGIONS

### DENTATE GYRUS GRANULE CELLS

A powerful tonic GABAergic signaling has been described in granule cells of the dentate gyrus already at P3 (Holter et al., 2010). Using subunit-specific pharmacological modulators, it has been demonstrated that, as in adults (Glykys et al., 2008), this conductance is mediated by a5 and d subunits containing extrasynaptic GABA_A_ receptors. The relative contribution of receptors containing α5-subunits decreases with age, while that of receptors containing δ-subunits increases, indicating that as in principal cells the expression of d subunits is developmentally regulated. The increased expression of d subunits parallels that of tonic conductance that reaches its maximum during the second postnatal week and then declines (Holter et al., 2010). Surprisingly, unlike immature CA1 and CA3 principal cells (Marchionni et al., 2007), this conductance exerts mainly an inhibitory effect* via *a**shunting inhibition. The inhibitory effect would prevent the occurrence of neonatal seizures (Holter et al., 2007).

### NEOCORTICAL NEURONS

A GABA_A_-mediated tonic conductance has been detected in layer V pyramidal cells of the somatosensory cortex of newborn animals (Sebe et al., 2010). This conductance, which is mainly mediated by a5 and d subunits containing extrasynaptic GABA_A_ receptors, is very large immediately after birth and then decreases dramatically during the second postnatal week. The developmental change appears to be related to the enhanced clearance of GABA from the extracellular space (due to the increased activity of GABA transporters) and to the reduced expression of δ subunits (the a5 subunits are expressed also in adults; Yamada et al., 2007). In cell-attach recordings GABA has been found to exert opposite effects on immature cell excitability, decreasing or increasing cell firing in accord with the value of *E*_Cl_ relative to resting membrane potential (Sebe et al., 2010).

Unlike the somatosensory cortex, in the visual cortex tonic GABA_A_-mediated currents increase with age (Jang et al., 2010). This may be related to the role of GABA in shaping neuronal circuits during a well-defined period of early postnatal development called critical period (Sale et al., 2010). In this period, the experience-dependent maturation of the visual acuity closely relies on the development of the intracortical GABAergic inhibition. Dark rearing, which causes a permanent loss of visual acuity reduces also GABAergic signaling mainly from parvalbumin positive interneurons (Hensch, 2005).

### CEREBELLAR GRANULE CELLS

The cerebellum is the first brain structure where a persistent background GABA_A_-mediated conductance has been characterized (Kaneda et al., 1995). This clearly exhibits a developmental profile, increasing progressively after P7. This may reflect the increasing number of GABAergic terminals within the glomerulus, a structure that limiting GABA diffusion may render the action of ambient GABA more efficient (Brickley et al., 1996; Wall and Usowicz, 1997). In addition, while at early developmental stages tonic GABA exerts a depolarizing and excitatory action, later it exerts an inhibitory effect *via* shunting inhibition (*E*_Cl_ is close to the resting membrane potential; Brickley et al., 1996).

### THALAMIC RELAY NEURONS

A similar sequence of events takes place in thalamic ventrobasal relay neurons, where GABA_A_-mediated tonic current is developmentally regulated. This conductance can be detected from the first week of postnatal life (Belelli et al., 2005). It progressively increases with age. This effect is not related to changes in the expression of GABA transporters, since bicuculline-induced shift in baseline current does not change by blocking GABA uptake with selective inhibitors (Peden et al., 2008). The ontogenetic up-regulation of the tonic conductance depends on marked changes in subunit composition of extrasynaptic GABA_A_ receptors with increased expression of α4 and δ subunits, as assessed with electrophysiology and immunohistochemistry. While in adult neurons, the persistent activation of extrasynaptic receptors by ambient GABA leads to a membrane hyperpolarization needed to promote low threshold burst firing (Cope et al., 2005), in neonates its function is not clear since it depends on the hyperpolarizing or depolarizing action of GABA.

### STRIATAL MEDIUM SPINY NEURONS

In GABAergic medium spiny striatal output neurons (MSNs), a tonic GABA_A_-mediated conductance starts appearing toward the end of the second postnatal week (Ade et al., 2008; Kirmse et al., 2008). Before P14, it can be detected only in the presence of GAT-1 inhibitors suggesting that this transporter operates in a net uptake mode, able to efficiently remove GABA from the extracellular space. An increase in synaptic GABA release, due to an up-regulation of the glutamatergic drive to MSNs may account for the persistent activation of extrasynaptic GABA_A_ receptors with age. Ambient GABA originates mainly from action potential-dependent synaptic release, since TTX reduces the tonic current similarly to GABA antagonists (Ade et al., 2008). The inhibitory GABA_A_-mediated conductance is larger in MSNs expressing dopamine D2 receptors, which project to the globus pallidus respect to those expressing dopamine D1 receptors which project to the substantia nigra pars reticulata (Ade et al., 2008; Santhakumar et al., 2010). While the first are supposed to inhibit movements, the latter to facilitate them (Gerfen et al., 1990). The greater expression of α5 subunit containing GABA_A_ receptors in D2- respect to D1-positive MSNs may account for the observed effects (Ade et al., 2008). In addition, using conditional b3 subunit knock-out mice Janssen et al. (2011) have demonstrated that receptors containing the b3 subunit also contribute to tonic inhibition in D2-positive neurons. These data are in agreement with previous mRNA expression studies showing that young but not adult striatal tissue primarily expresses α2, α5, β3, and γ GABA_A_ receptor subunits (Laurie et al., 1992). Differences in tonic currents between D1- and D2-positive cells observed in juvenile animals may be crucial for regulating MSNs excitability and motor output.

## CONCLUSION

From the data reviewed here it is clear that a tonic GABA_A_-mediated conductance plays an important role in brain development. This conductance is developmentally regulated and differs in various brain regions (see **Table 1**). While in some areas (i.e., hippocampus and somatosensory cortex) it diminishes with maturation, in others (i.e., thalamus, visual cortex, striatum, and cerebellum) it increases. This depends on several factors including stage of cell maturity, geometry of the synapses, interneuronal firing, neurotransmitter diffusion, expression and distribution of extrasynaptic GABA_A_ receptors, and/or GABA transporters. In addition, according to the direction of GABA signaling, accumulation of the neurotransmitter in the extracellular space may differently affect network excitability.

**Table 1:** Tonic GABAergic currents in various brain regions during early development.

Structure	Age	GABA_A_ R subunits	Direction of GABA action	Reference
CA1 region of hippocampus	Late embryonic, early postnatal life (1st week)	*α*5, *γ*2	Depolarizing	Demarque et al. (2002)
				Marchionni et al. (2007)
CA3 region of hippocampus	1st postnatal week	α1, α3, *α*5, β3, *γ*2	Depolarizing	Marchionni et al. (2007)
				Sipilä et al. (2007)
Dentate gyrus	Newborn cells in adulthood	Unknown	Depolarizing	Ge et al. (2006)
Somatosensory cortex	1st postnatal week	α5, δ	Depolarizing	Sebe et al. (2010)
Cerebellum	1st postnatal week	Unknown	Depolarizing	Brickley et al. (1996)
	2nd postnatal week	Unknown	Hyperpolarizing	Wall and Usowicz (1997)
Thalamus (ventrobasal)	1st postnatal week	α4, δ	Unknown	Peden et al. (2008)
Striatum	2nd postnatal week	α5	Unknown	Ade et al. (2008)
	4th postnatal week	δ, β3	Unknown	Santhakumar et al. (2010)
				Janssen et al. (2011)

In spite progress in the field much remains to be known on how early activity or experience regulates receptors trafficking and exchanges between synaptic and extrasynaptic pools. While the interaction of scaffold proteins with synaptic receptors has been at least in part elucidated that related to extrasynaptic receptors is still poorly understood. Furthermore, it would be of interest to know how selectively silencing in embryonic or early postnatal life a particular extrasynaptic GABA_A_ receptor subunit may alter the computational properties of neuronal circuits and synaptogenesis. This would help to better understand the role of GABA as a developmental signal and its implications in neurodevelopmental disorders.

## CONTRIBUTION

Giada Cellot and Enrico Cherubini wrote the paper.

## Conflict of Interest Statement

The authors declare that the research was conducted in the absence of any commercial or financial relationships that could be construed as a potential conflict of interest.
